# Prompt improvement of a pressure ulcer by the administration of high viscosity semi-solid nutrition via a nasogastric tube in a man with tuberculosis: a case report

**DOI:** 10.1186/1752-1947-4-24

**Published:** 2010-01-27

**Authors:** Tamaki Nakayama, Seiji Hayashi, Kyoichi Okishio, Tomoko Tomishiro, Kaori Hosogai, Yuki Ootsu, Yasushi Morioka, Kazuyoshi Hatsuda, Eriko Naito, Mitsunori Sakatani

**Affiliations:** 1 National Hospital Organization Kinki-Chuo Chest Medical Center, Nagasone-Cho, Kita-Ku, Sakai-City, Osaka, 591-8555, Japan

## Abstract

**Introduction:**

Semi-solid nutrition with high viscosity has the advantage of reducing gastroesophageal reflux and diarrhea and shortens the duration of administration compared with liquid nutrition. This is the first report describing the administration of semi-solid nutrition with high viscosity via a nasogastric tube, which achieved a remarkable improvement in the patient's nutritional state.

**Case presentation:**

A 67-year-old man (mongoloid race, Japanese) with tuberculosis, a pressure ulcer and malnutrition was admitted to our hospital. He also had right hemiplegia, dysphagia and aphasia as sequelae of a cerebral hemorrhage. Before his admission, he had been treated at another hospital with 600 kcal/day of liquid nutrition via a nasogastric tube, which was insufficient and induced severe malnutrition. After he was admitted to our hospital, we increased the quantity of his liquid nutrition without success because of complications, specifically diarrhea and gastroesophageal reflux. As it was difficult to confirm whether or not he would accept gastrostomy feeding, we administered semi-solid nutrition with high viscosity (20,000 mPa x s) via a large-bore nasogastric tube (18 French). Soon after he was started on semi-solid nutrition, his pressure ulcer and malnutrition improved without diarrhea or complications accompanying the large-bore nasogastric tube.

**Conclusion:**

When patients have problems with liquid nutrition, such as diarrhea or gastroesophageal reflux, semi-solid nutrition via a nasogastric tube is a useful method of achieving improvements in nutritional state in a short period of time.

## Introduction

Enteral nutrition has surpassed parenteral nutrition in terms of safety and physiological benefits [1,2]. For a patient who has problems swallowing but has an intact intestinal tract, enteral nutrition is primarily recommended [1]. Semi-solid enteral nutrition has the advantage of lowering the risk of diarrhea and esophageal reflux [3]. Here we report a case in which malnutrition, diarrhea and a pressure ulcer were improved by high viscosity semi-solid nutrition via a large-bore nasogastric tube.

## Case presentation

A 67-year-old man (mongoloid race, Japanese) was admitted to our hospital because of a 3-day history of fever. Acid-fast bacilli was found to be smear-positive in his sputum, and a chest radiograph and computed tomography examinations showed parenchymal opacities with scattered fine nodules in his right apical region. He was diagnosed with pulmonary tuberculosis, so he was started on anti-tuberculosis therapy with isoniazid, rifampicin, ethambutol, and pyrazinamide on the first day that he was hospitalized. He had developed a cerebral hemorrhage 18 months before this hospitalization, and he had right hemiplegia, dysphagia, and aphasia as sequelae. He had been receiving 600 kcal/day of liquid nutrition via a nasogastric tube for 6 months before the current hospitalization.

**Figure 1:**
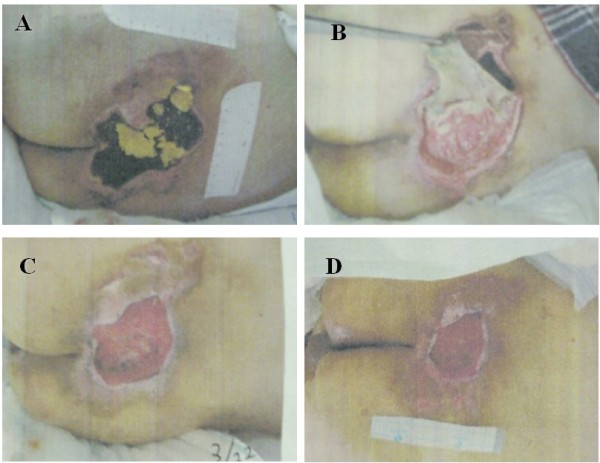
**Chronological change of pressure ulcer**. **(A) **Day 0. The patient had a 16.5 cm × 15.5 cm, grade IV pressure ulcer in the sacral region. **(B) **Day 22. Debridement was conducted. **(C) **Day 66. The pressure ulcer had shrunk along with an improvement in the patient's state of nutrition. **(D) **Day 93. The size of the pressure ulcer was 8.0 cm × 5.0 cm.

**Figure 2:**
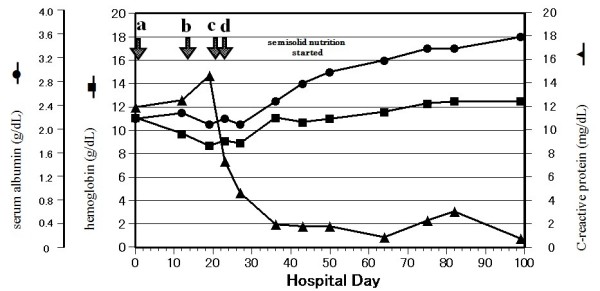
**Chronological change of hemoglobin concentration (square), serum albumin concentration (circle) and C-reactive protein titer (triangle)**. Anti-tuberculosis drugs were started on the first day of admission (arrow a). Liquid nutrition was increased to 1,200 kcal/day on the 14^th ^day of admission (arrow b). Semi-solid nutrition of 1,200 kcal/day was started on the 21^st ^day of admission (arrow c). Debridement of the sacral pressure ulcer was conducted on the 22^nd ^day of admission (arrow d).

On admission to our hospital, he had a 16.5 cm × 15.5 cm, grade IV [4] pressure ulcer in the sacral region, from which *Escherichia coli *and methicillin-resistant *Staphylococcus *were detected (Figure 1, panel A). His albumin count was 2.2 g/dL, hemoglobin was 11.1 g/dL, C-reactive protein was 12.0 mg/dL (Figure 2) and his body temperature was 38°C. A liquid nutrition of 200 kcal was administered for 60 minutes, 3 times a day, and this regimen was continued for 2 weeks. In order to improve his state of nutrition and to reduce weight-bearing on the sacral region, 1,200 kcal/day of liquid nutrition was administered for a shorter time. This, however, induced watery diarrhea and gastroesophageal reflux. His general condition and malnutrition (low serum albumin) suggested that he would have a poor prognosis if a gastrostomy was performed [5]. It was difficult to confirm whether or not he would accept gastrostomy feeding, so nasogastric tube feeding was continued. As for the preparation, we selected semi-solid nutrition with higher viscosity (20,000 mPa × s). In order to achieve the administration in a certain short period of time, a nasogastric tube of 18 French was inserted.

As the patient was febrile and bedridden, his total energy expenditure was assessed as 1,708 kcal/day by the Harris-Benedict equation [6] (presumed height 162 cm, presumed body weight 45 kg, ideal body weight 57.7 kg, activity factor = 1.1, stress factor = 1.5). Considering that his caloric prescription up to that time had been 600 kcal/day and he had diarrhea, we first tried 1,200 kcal/day.

On day 21 in hospital, a semi-solid enteral product of 400 kcal/267 g (PG Soft™, Terumo, Tokyo, Japan) was administered for 15 minutes 3 times a day, which was then followed by 250 mL of semi-solidified water (PG Water™, Terumo, Tokyo, Japan) and dietary fiber. After starting the semi-solid enteral product, he experienced no diarrhea or esophageal reflux. On day 22, a debridement of the sacral pressure ulcer was conducted. Four weeks later, an improvement was observed in his albumin, hemoglobin, and C-reactive protein levels (Figure 2). His pressure ulcer was then 8.0 cm × 5.0 cm (Figure 1, panel D).

No complication of the esophagus, paranasal sinus, or nose wings accompanying insertion of the nasogastric tube was observed. Compliance of the large-bore nasogastric tube was favorable, and he did not try to remove the tube himself. With continuous maintenance of the tube, no obstruction was observed. Administration of anti-tuberculosis drugs was continued via the nasogastric tube without any adverse effects, and tubercle bacillus was not detected in his sputa. After 3.5 months, he was transferred to another facility for further recuperation.

## Discussion

Our case report showed that nutrition improvement and curative effects were obtained by nutrition with a higher viscosity of about 20,000 mPa × s. In recent years, the advantages of semi-solid nutrition over liquid nutrition have been reported [3]. Liquid nutrition may not be the best choice when it is needed to shorten the administration time and at the same time provide a sufficient amount of calories. A small-bore nasogastric tube is thus recommended from a viewpoint of compliance. However, in this case, liquid nutrition was not appropriate, and so we had no choice but to use a large-bore nasogastric tube.

Due to its higher viscosity, semi-solid nutrition has several advantages. One advantage is the reduction of gastroesophageal reflux with the resultant prevention of aspiration pneumonia [3]. The rate of proximal stomach emptying contributes to the number of reflux episodes per hour [7]. Nutrition with higher viscosity promotes the passage of gastric content to the intestinal tract, which shortens the gastric retention time [3,8]. Another advantage is the prevention or improvement of diarrhea. We used semi-solid nutrition for patients with intractable diarrhea who had been administered with liquid nutrition. Improvement of diarrhea was observed in 9 out of 14, or 64.2% of patients (unpublished data). The third advantage is that semi-solid nutrition can shorten the duration of administration. The bolus administration of liquid nutrition induces gastroesophageal reflux. Hence, in order to prevent the reflux, continuous administration is required [9]. The manufacturer of the nutrition we used for this patient recommends that the administration of the semi-solid nutrition be done in 15 minutes. Administration should be carried out in a sitting position to minimize gastroesophageal reflux, but this position may impose load to any pressure ulcer at the sacral region.

In order to administer semi-solid nutrition in a short time, we changed the size of our patient's nasogastric tube from small-bore to 18 French, and the pressure ulcer that had existed for a long time disappeared in 3 months. As for amelioration of the pressure ulcer, the favorable effects of debridement and the antibacterial effect of anti-tuberculosis drugs must be taken into consideration. However, it is established that nutrition improvement is indispensable for the amelioration of a pressure ulcer [10,11]. Hence, we believe that shortening the administration time of nutrition largely contributed to the improvement of our patient's pressure ulcer.

It is not recommended to administer semi-solid nutrition through a thinner tube with high pressure because the liquid spouting from the tip may injure the gastric mucosa. When semi-solid nutrition is given via a nasogastric tube, there is a risk for tube obstruction, but we avoided this by using a large-bore tube.

## Conclusions

When patients have problems with liquid nutrition, such as diarrhea or gastroesophageal reflux, semi-solid nutrition via a nasogastric tube is a useful method for improving nutrition in a short period of time.

## Consent

Written informed consent was obtained from the patient for publication of this case report and any accompanying images. A copy of the written consent is available for review by the Editor-in-Chief of this journal.

## Competing interests

The authors declare that they have no competing interests.

## Authors' contributions

TN, KH, YO and YM performed the assessment of the nutrition state and designed the nutrition treatment plan of the patient. SH supervised the nutrition treatment plan, wrote the manuscript, and reviewed the international literature. KO, TT and EN treated the patient's tuberculosis and pressure ulcer. KH participated in the assessment of the patient's clinical data. MS supervised the treatment of tuberculosis and revised the manuscript for important intellectual content. All authors read and approved the final manuscript.
